# Antibodies to group A streptococcal virulence factors, SIC and DRS, increase predilection to GAS pyoderma

**DOI:** 10.1186/s12879-015-0857-4

**Published:** 2015-03-03

**Authors:** Mohan G Karmarkar, Gouri P Hule, Ainslie Cameron, Preeti R Mehta, Uday Khopkar, Niwrutti K Hase, Kadaba S Sriprakash

**Affiliations:** KEM Hospital, Mumbai, India; QIMR Berghofer Medical Centre, 300, Herston road, Brisbane, QLD 4006 Australia; Present Address: Griffith University, Gold Coast Campus, Gold Coast, QLD Australia

**Keywords:** Streptococcus pyogenes, Post streptococcal glomerulonephritis, Pyoderma, Streptococcal inhibitor of complement

## Abstract

**Background:**

*Streptococcus pyogenes* (group A streptococcus; GAS) is an etiological agent for pharyngitis, pyoderma, and invasive infections in humans. Pharyngitis and pyoderma may lead to serious immune sequelae such as rheumatic heart disease and post-streptococcal glomerulonephritis (PSGN). Streptococcal Inhibitor of Complement (SIC) and its orthologue, distantly related to SIC (DRS), are virulence factors expressed by only four of more than 100 M types of GAS. These four types (M1, M57, M12 and M55) are among the M types, which are associated with PSGN. In several populations PSGN has been shown to be a risk factor for chronic kidney disease (CKD) and end-stage renal disease (ESRD). Previous studies showed SIC or DRS antibody-prevalence was associated with PSGN, and seroprevalence of SIC antibodies is significantly high among CKD and ESRD patients in Mumbai.

**Methods:**

Streptococcal isolates recovered from GAS pyoderma cases were typed. Seropositivity for SIC and DRS antibodies in subjects with pyoderma, PSGN pediatric cases, age matched healthy controls and non-GAS pyoderma cases were determined.

**Results:**

We confirm in this study an association between seroprevalence to SIC and DRS antibodies, and PSGN in Mumbai population despite low point prevalence of M1, M12, M55 and M57. In addition we extended the study to GAS-pyoderma and non-GAS pyoderma cases. To our surprise, we found a positive association between the seroprevalence to SIC and DRS antibodies, and GAS-pyoderma owing to infection with diverse M types. The mechanism of increased predisposition to pyoderma owing to infection by diverse GAS among SIC or DRS antibody-positive population is not clear. Nonetheless, our findings could be explained by a phenomenon akin to antibody-dependent enhancement (ADE).

**Conclusions:**

This is the first report showing a small number of GAS M types conferring predisposition to pyoderma by diverse types. Implications of this ADE-like phenomenon are discussed in the light of evolutionary advantage to GAS, vaccine design and control of renal diseases.

## Background

*Streptococcus pyogenes* (group A streptococcus; GAS), a human-specific pathogen, is responsible for diverse diseases such as pharyngitis, pyoderma, cellulitis, necrotising faciitis, toxic shock syndrome and life-threatening immune sequelae including rheumatic heart disease and post-streptococcal glomerulonephritis (PSGN) [1,2]. Pharyngitis and pyoderma caused by some GAS strains (*emm* types) are associated with PSGN, of which *emm*1, 12, 55, 57 are among the major ones. These *emm* types express a major secretory antigen called Streptococcal Inhibitor of Complement (SIC; in *emm1* and *emm57*) or its orthologue called distantly related to SIC (DRS; in *emm12* and *emm55*) [3-5]. Although Sagar et al. [6] reported that SIC was expressed by isolates of *emm1-2* type which was claimed to be distinct from *emm1* subtypes, isolates of both *emm1* and *emm1-2* are clonally related. Both SIC and DRS elicit high antibody responses during natural infections and the antibodies are persistent [7,8].

Serological reactions to several GAS antigens have been observed subsequent to PSGN [9]. In an earlier community-based study seropositivity to DRS, but not to SIC, was found to be associated with history of PSGN among the Indigenous Australians [8]. Subsequently a Swedish hospital-based study found an association between acute PSGN cases and IgM antibodies to SIC in sera from pediatric cases [10]. The apparent differences in the above results could be because of differences in the distribution of *emm* types in these two geographical regions, to the differences in study design or both. Although the prognosis of PSGN is generally excellent, many studies suggest that PSGN is a strong risk factor for chronic kidney disease (CKD) and end-stage renal disease (ESRD) [11-14]. Interestingly we recently showed [15] that SIC antibody seroprevalence is higher in CKD and ESRD patients than in control subjects in Mumbai, a region endemic for streptococcal diseases. Furthermore we found that anti-SIC seropositivity in CKD patients may result in poor prognosis, the disease progressing to ESRD. These findings warranted a hospital-based investigation of association between SIC and DRS antibody-prevalence and PSGN in Mumbai area.

We now show that acute PSGN pediatric patients have high seroprevalence for SIC and DRS antibodies. We also extended this study to pyoderma patients attending outpatient wards of the same hospital. To our surprise we found that significantly greater proportion of GAS pyoderma patients are positive to SIC and DRS antibodies than those with non-GAS pyoderma patients, or age-matched healthy control subjects. Despite this observation, SIC or DRS positive *emm* types were not overly represented among the isolates from the GAS pyoderma cases. This startling finding clearly highlights increased predisposition to GAS pyoderma in Mumbai region among the subjects seropositive to SIC or DRS antibodies due to past infection with *emm* types expressing these antigens. We attribute this to a phenomenon akin to antibody-dependent enhancement (ADE) of skin infection. However, only a limited number of GAS strains seem to confer ADE of infection by diverse GAS types. Such ADE may have a role in the evolution of GAS as a highly successful human pathogen. We discuss implications of our findings in relation to the management of CKD patients and vaccine strategy.

## Methods

### Subjects, swabs and blood collection

Informed consent was obtained from all participants and guardians. The study was conducted under ethics committee approval from Seth G.S. Medical College & KEM Hospital (reference number, EC/GOVT-4/2010). All the patients and control subjects are of similar demography. All the subjects included were from low socio-economic strata living in vicinity of KEM Hospital and the study was conducted in 2 years between June 2011 and June 2013. They were therefore temporally and geographically matched populations. The control 1 subjects were age-matched to that of PSGN patients; they are paediatric population. The control 2 subjects and the cases of non-GAS pyoderma (caused by *Staphylococcus*) were age matched to that of GAS pyoderma cases (mainly adult population).

Swabs from 701 patients attending skin-outpatient department at KEM hospital were collected during 2 years of the study period. GAS isolates were recovered from 150 subjects with primary GAS pyoderma diagnosed as impetigo, superficial folliculitis, ecthyma and furuncles. Multiple isolates were available from the same swab for 20 cases among the pyoderma cases. For further 17 cases while mixed infections with GAS and *Staphylococcus* were found in some skin sores, no multiple strains of GAS was recovered from a patient.

Blood samples for sera were collected from a) the above 150 GAS pyoderma subjects (mean age 25.1 ± 17.09 years; range 2–48 years) at the time of their visit to the hospital’s outpatient department, and b) 25 children with PSGN (mean age 7.12 ± 3.13 years; range 1–11 years) admitted to the pediatric ward of KEM hospital diagnosed as acute nephritic syndrome and having ASO titres > 300 IU (see below).

Blood samples were also collected from 50 non-GAS pyoderma cases (mean age 25 years; range 2–37 years) and two age-matched control groups (control group 1 for PSGN [n = 25; mean age 6.6 years]; and control group 2 for pyoderma [n = 50; mean age 25.8 years]). Sera samples from patients and healthy individuals were stored at −80°C until used.

### GAS *emm* typing

The isolates were characterized by *emm* typing according to the protocol described at www.cdc.gov/ncidod/biotech/strep/strepblast.htm.

### Serology

Anti-streptolysin O (ASO) titers in sera were determined according to Karmarkar et al. [16]. Both control groups had no known history of streptococcal infections. ASO titers were <300 IU for control groups. Non-GAS pyoderma are ASO negative.

Serum antibody titres for SIC and DRS were measured by Enzyme-Linked Immunosorbent Assay (ELISA) as described by Karmarkar et al. [15]. Briefly, high binding flat bottomed immune plates (Himedia) were coated by adding 100μl of recombinant his-thioredoxin-tagged SIC, DRS or recombinant his-tagged thioredoxin (100 μg/ml; in carbonate buffer, pH 9.6) per well and incubating overnight at 4°C. After blocking with denatured casein hydrolysate (5% in PBS) for 2 hours, the bound protein was allowed to react with a 1 in 300 dilution of human serum for 1 hour in the same buffer containing 0.05% Tween-20 at room temperature. After washing 5 times with 0.05% Tween-20 in PBS, secondary anti-human IgG antibody conjugated with peroxidase (Sigma-Aldrich) was added and the reactions were detected with 3,3′,5,5′-tetramethylbenzidine (Sigma-Aldrich). The reaction was terminated with stopping solution after 30 min incubation and optical density (OD) was measured at 450 nm. Seropositivity or seronegativity was determined by using cutoff values as described below (see statistical analysis). Experimental controls with no antigen, recombinant his-tagged thioredoxin, no sera, and no secondary antibodies were included.

### Competitive ELISA

Serum samples (1 in 300 dilution in PBS, pH 7.4) were pre-incubated at 37°C with competitor antigens for 1 hour before adding to plates coated with SIC or DRS. The competing antigens were: a) no antigen (control), b) 10 μg of SIC, DRS or both, c) 50 μg of SIC, DRS or both. The rest of ELISA conditions were as described above.

### Urinalysis

Urine was collected for all PSGN cases and the control group 1. Proteinuria, protein-creatinine ratio, blood cells in urine were determined as described earlier [17,18].

### Statistical analysis

Cutoff values to assess whether or not a serum sample should be regarded as positive for SIC or DRS antibody were determined as before [15] and were obtained by taking two standard deviations above the mean OD values for control groups for SIC and DRS separately. Sera samples with OD values above the cutoff are scored as positive. Statistical significance was calculated by unpaired t-test.

## Results

### Heterogeneity of strains recovered from pyoderma cases

Of the 701 patients, GAS isolates were recovered from 150 pyoderma cases. Multiple isolates from the same skin sore for each subject were analyzed for 20 pyoderma patients, and in an additional 17 pyoderma cases isolates from multiple skin sores were *emm* typed. In all, the isolates belonged to 57 *emm* types or sub-types over the 2 year study period, and each type is represented by 1–5 isolates in a year (Table 1). Hence there was no evidence for expansion or outbreak of a particular type during this study. Of the 150 isolates only 7 (4.7%) were *emm*1 and *emm*12 which express SIC and DRS respectively (marked by # in Table 1); *emm55* and *emm57* were not recovered. Despite high diversity, multiple GAS isolates from patients (different colonies from the same swab or different swabs; n = 37) were of the same type for each patient. Taken together our results show it is unlikely that SIC- or DRS-positive types *per se* are main contributors to pyoderma in these subjects; instead diverse *emm* types circulating in the community are responsible for skin infection.

**Table 1:** **Types and subtypes recovered in the two years of study period**

**Year 1**		**Year 2**	
**emm types**	**Recovered from number of patients**	**emm types**	**Recovered from number of patients**
emm63.3	3	emm78.3	5
emm82.1	3	emm116.1	3
st9505	3	emm122	5
emm42.3	3	emm 25.2	3
emm22	3	emm 53*	5
emm49.4	4	emm 97.1	2
emm15*	2	emm 227.1	4
emm106	2	emm85.0	5
emm12#	3	emm66*	3
emm53.9	2	emm11.8	3
emm73	2	emm8.1	3
emm86.2	4	emm1*#	3
emm119.2	2	emm106	3
st11014	2	emm58.8	3
st854	2	emm56	1
emm 109.1	2	emm15*	1
emm53*	2	emm49	2
emm15.1	1	emm 112	3
emm60.3	1	emm 4	4
emm66*	1	emm 100	3
emm73.1	1	emm 105	1
emm9	1	emm 111	1
st1731.3	1	emm 102	1
stKNB6	1	emm108	1
emm1*#	1	emm77	1
emm3	1	emm113	2
emm118.5	1	St1389	1
		emm75	5
		emm69	4
		emm36	5
		emm25	3
		emm11	3
		emm82	2
		emm28	2

The strains indicated by # represent SIC or DRS positive types. Types indicated by asterisks were recovered in both years.

### Anti-SIC and anti-DRS seroprevalence

**Figure 1:**
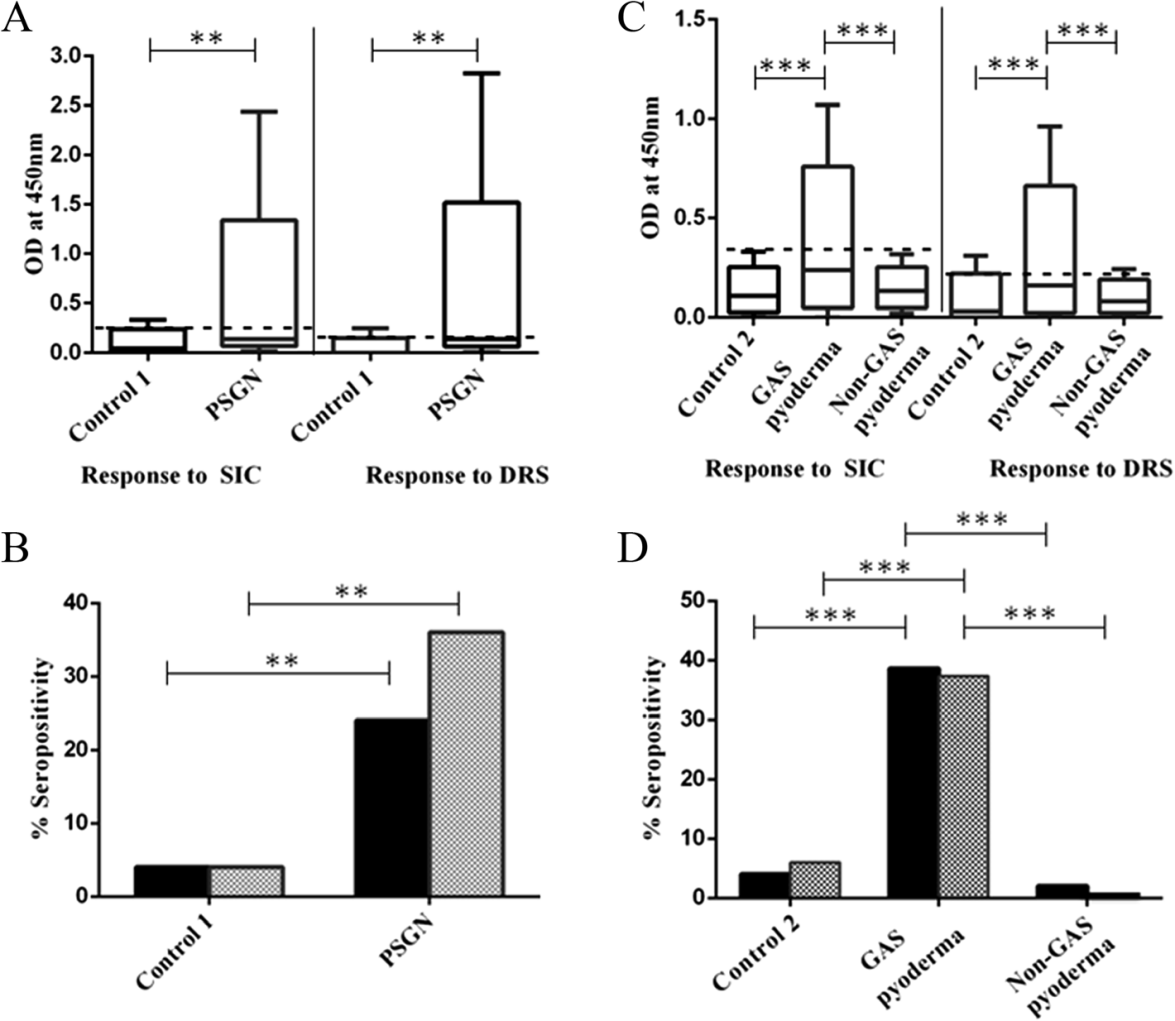
***Seroprevalence for SIC and DRS antibodies in PSGN, pyoderma and control groups.*** Recombinant SIC and DRS proteins with a thioredoxin tag were used as the streptococcal antigens for ELISA. Background optical density (OD^450^) readings to thioredoxin were subtracted from all ODs. OD values for control 1 and PSGN cases (n = 25 each) are shown in panel **A** and for control 2 (n = 50), GAS pyoderma (n = 150) and non-GAS pyoderma (n = 50) cases in panel **C**. The box-whisker plots show median (cross bar in the box), quartiles, and range. The mean OD values for control 1 are 0.081 and 0.042 for SIC and DRS respectively; and those for control 2 are 0.12 and 0.076 respectively. These means and those for non-GAS pyoderma cases (0.14 and 0.09 for SIC and DRS respectively) are not significantly different. The means for PSGN and GAS pyoderma cohorts are significantly higher than their controls for both SIC and DRS (p < 0.05, >0.01 for PSGN; and p < 0.0001 for GAS pyoderma). The calculated cutoff values (mean + 2x Standard deviation for the corresponding control) is shown as dotted line across. Samples scoring equal to or above the cutoff values were scored as positive. Panels **B** and **D** show per cent seropositive samples for SIC antibodies (black bars) or DRS antibodies (shaded bars) in each group and compared with respective controls. Lines with asterisks indicate statistically significant differences (** = 0.05 ≤ p > 0.0001; *** = p ≤ 0.0001) between the means in **1**A and **1**C, and between proportion of seropositives in 1B and 1D. Lack of such lines means there is no significant differences between groups (eg; control 2 and non-GAS pyoderma groups in **1**C and **1**D).

Sera from a) the 150 GAS pyoderma subjects, b) 25 children with PSGN, c) 50 non-GAS pyoderma cases, d) two age-matched control groups; control 1 for PSGN and control 2 for GAS pyoderma were analysed for various parameters. Both the control groups and the non-GAS pyoderma cases had no known history of streptococcal infections, and ASO titres were <300 IU. This suggested no recent GAS infection in these individuals. Furthermore, urinalysis of the control 1 showed no proteinuria, protein-creatinine ratio <0.2, and absence RBCs or pus cells suggesting no subclinical PSGN or CKD in this control group. Figure 1A shows optical density values for SIC and DRS antibodies, median and quartiles for control 1 and PSGN cases. The mean values for PSGN are significantly higher than those of the control (p < 0.05, >0.01; Figure 1 legend). The calculated cutoff values (mean + 2× standard deviation of the corresponding control) which are used to determine seroprevalence, are shown in Figure 1A and C (dotted lines).

The low recovery rates of SIC- and DRS-positive types (*emm*1 and *emm*12) among the pyoderma cases in the 2 years (4.7%, see above) may simply be a reflection of low prevalence of these types circulating in the KEM hospital catchment-community. Since SIC and DRS antibodies are persistent [7,8], we would expect rates of seroprevalence for these antibodies to be commensurate with the low abundance of these types. In accordance with this, we observed that 4-6% of the control 1 (also in control 2 and non-GAS pyoderma; see later) was seropositive to SIC, DRS or both (Figures 1B). By contrast, antibody seroprevalence rates (proportion greater than mean + 2SD of control 1) in the PSGN patients were significantly high; 24% and 36% for SIC and DRS respectively (Figure 1B; correspondingly p = 0.0279; p = 0.0297). Odds ratio (OR) for SIC and DRS antibody positivity are 7.56 (95% CI 0.84, 68.46; p = 0.07) and 13.5 (95% CI 1.56, 117.14; p = 0.0182) respectively. Our results in Mumbai population, which is highly endemic for streptococcal diseases, are concordant with earlier observations from the Australian Indigenous population and Swedish pediatric cases [8,10], and thereby provide further support to the observation of positive association between seroprevalence of these antibodies and PSGN.

We then extended seroprevalence study to GAS pyoderma cases and compared to age matched control 2 and non-GAS pyoderma cases (Figure 1C and D). It is interesting that mean values for control group 2 and non-GAS pyoderma are not significantly different with each other or with control 1 (see Figure 1 legend), despite the differences in mean age of the two healthy control groups. Together, the data for the two control groups and the non-GAS pyoderma cohort establish base-level seroprevalence rate of <5% for SIC or DRS antibodies in this population (Figure 1B and D); and this rate is in agreement with the recovery rates of SIC- or DRS-positive *emm* types in the community (Table 1).

Unexpectedly as shown in Figure 1D, 58 (38.7%) of GAS pyoderma patients are anti-SIC positive and 56 (37.3%) are anti-DRS positive. These proportions are significantly higher (p <0.0001 for each) than for the corresponding control group (control 2). The OR for SIC is 15.13 (95%CI 3.54, 64.64; p =0.0002) and for DRS is 9.33 (95%CI 2.77, 31.40; p =0.0003).

**Figure 2:**
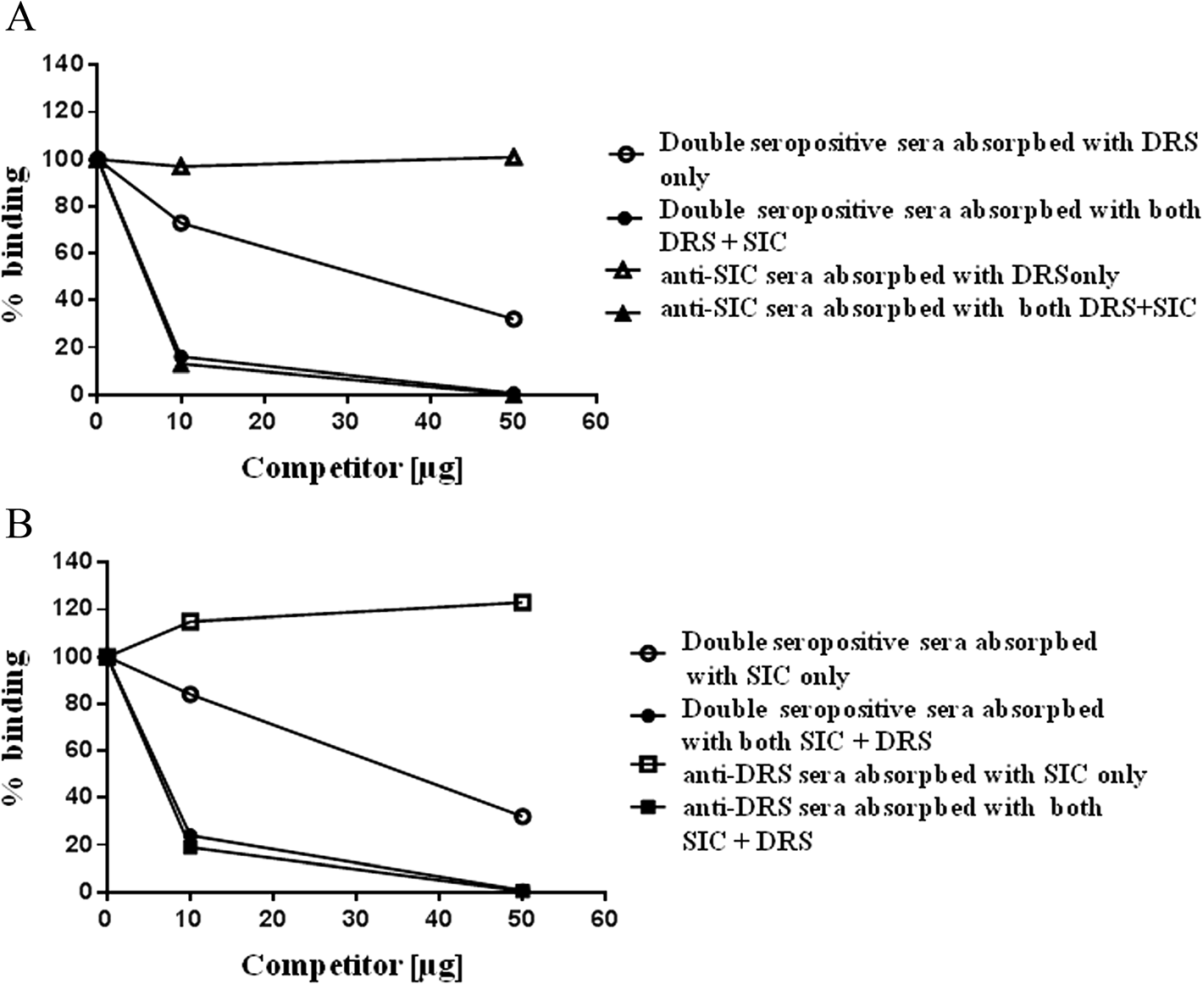
***Competitive ELISAs.*** Sera samples positive to SIC only, DRS only or to SIC + DRS were pre-incubated with homologous, heterologous or both competitor antigens at 0 μg, 10 μg and 50 μg prior to incubation with SIC **(Panel A)** or DRS **(Panel B)** coated plates for ELISA.

Interestingly, the proportion of the samples reacting to both SIC and DRS antigens was found to be 28.66% in the pyoderma cases. This is significantly higher than in the control group 2 (p = 0.0051; Chi square). The fact that some patients exhibit seroreaction to one or the other antigen, suggests that double seropositivity is not due to possible cross-reactions of the antibodies with the antigens. Moreover, mature SIC and DRS proteins have only limited homology; mostly confined to the proline-rich region [5]. To confirm the true nature of the double seropositivity, we carried out competitive ELISAs on a) 6 sera showing reactions to both antigens, b) 4 sera showing reactions to SIC only and c) 4 sera showing reactions DRS only. While all the 8 sera showing reactions only to SIC or DRS can be competed out by pre-incubating the sera with homologous but not heterologous antigen, the 6 sera reacting to both SIC and DRS required both the antigens for full competition. Results with representative sera for each category are shown in Figure 2. These findings confirm that the samples with double seropositivity are not artifacts, but represent true results.

Since seroprevalence to these antibodies in non-GAS pyoderma cases, like in the control group 2, is significantly lower than in GAS pyoderma cases (p < 0.0001 for SIC and DRS), we believe this phenomenon of high SIC and DRS antibody seroprevalence is specific to GAS pyoderma cases.

## Discussion

SIC and DRS antibodies are persistent, and overwhelming evidence show that the distribution of the SIC and DRS genes is restricted to mainly four *emm* types [7,8,19]. The isolation rate of these four types from our pyoderma cohort is low (<5%), and our previous study from this region also show that these types are recovered in low numbers in Mumbai population [15]. Given these scenarios, this current observation of high seroprevalence rates to SIC and DRS antibodies specifically in patients with GAS pyoderma caused by many circulating types is indeed startling. Clearly it points out to increased predisposition to GAS pyoderma among the subjects who are seropositive to SIC or DRS antibodies owing to past exposure to *emm* types expressing these antigens. We attribute this to a possible role of SIC and DRS antibodies in a mechanism akin to antibody-dependent enhancement (ADE) of infection by GAS. While ADE of infection is common occurrences in viruses, bacteria too could deploy such strategies. For instance, ADE of infection has been reported in *S. pneumoniae* [20,21]. Cleavage of IgA1 by bacterial IgA1-protease enhanced adherence of *S. pneumoniae* to respiratory epithelial cells. More recently ADE-like phenomenon was observed in *Pseudomonas aeruginosa* wherein increased severity of respiratory infections was found to be associated with elevated levels of IgG2 against the bacterial O antigen [22]. The ADE-like phenomenon we observed here is different in an important way to what is known in other pathogens; only persistent antibodies to SIC or DRS owing to past infection by GAS strains expressing these antigens seem to confer ADE on all strains.

The seroprevalence rate is 38.7% for SIC antibodies in GAS pyoderma cases, and that for DRS antibodies is 37.3% (Figure 1D). Therefore, we would expect approximately 14% (38.7% of 37.3%) of the patients to show seropositivity to both the antibodies. However, we observed 28.66% of the cases were positive to both SIC and DRS antibodies - twice the expected frequency (the observed-to-expected ratio of ~2). A likely explanation for this observation is that SIC or DRS antibody mediated ADE predisposes to skin infections by many GAS strains including the SIC or DRS positive types. The above observed-to-expected ratio of 2 suggests that the rate of enhancement of infection due to SIC or DRS antibodies may be in the vicinity of two.

Further work is warranted, and being planned, to demonstrate the SIC and DRS mediated ADE-like phenomenon in pharyngeal infections. Also, demonstration of the ADE of infection in cell culture or in animal models may provide further support to this phenomenon. If ADE of GAS infections occur at both the major tissue sites, it may offer continued stability for the host-pathogen interaction in evolutionary terms, and may be important factor in GAS’s success as a human-specific pathogen. We suggest that the four SIC and DRS positive types (*emm* 1, 12, 55 and 57) are “core types” and the rest “non-core types”. Global distributions of *emm* types clearly show, whereas in most regions one or more of the 4 “core types” are recovered, the distributions of the “non-core types” vary considerably with some types predominating in many places and others are rarely found or not at all [23,24]. This suggests that it is possible that there are differences in infectivity among the *emm* types. Should the differences persist, it is possible that contributions to the GAS genetic pool by the low-infective types would be depleted at the expense of a few expanding types. ADE by the “core” types may partly correct this imbalance. Such a scenario may offer a possible novel strategy for efficient control of GAS diseases by targeting future vaccine candidates against the “core types”. By such intervention, a decrease in overall GAS infection may be achieved.

In many populations PSGN is an independent risk factor for CKD [11-14]. We recently showed that greater proportion of CKD and ESRD patients was positive to SIC antibody than that of healthy controls in the Mumbai population. However, no association between DRS antibodies and CKD or ESRD was found [15]. Taken together our studies show that whereas both SIC and DRS seropositivity may predispose to pyoderma through an ADE-like mechanism, and consequently result in increased seroprevalence in the PSGN patients, only the SIC antibody-positive PSGN patients have greater predilection to CKD and ESRD. Hence we suggest regular monitoring of children positive for SIC antibodies for signs of stage 1 CKD may provide an opportunity to offer proactive treatments early, so progression to ESRD may be averted or delayed.

## Conclusions

The role of SIC and DRS is not well understood. It is intriguing that only four types express these antigens. They elicit persistent antibody response. Therefore, antibody prevalence in a population is cumulative result of past infections by one of these four “core” types. Intuitively, one would expect that the rate of prevalence would be the same in a given population among various subjects; namely subjects with pyoderma, non-GAS pyoderma, PSGN cases or healthy controls. However, contrary to this expectation we found that the rate of seroprevalence is high specifically in GAS pyoderma cases. Since the pyoderma is resultant of diverse GAS strains not necessarily expressing these antigens, we interpret the results to mean that antibodies to SIC or DRS predispose subjects to skin infection by GAS. This is a unique phenomenon of ADE of infection rendered by a small number of GAS strains on a wide number of strains. Such a mechanism could have implications to maintenance of GAS gene pool despite possible inherent variations in infectivity.
